# The Impact of Antiretroviral Therapy in a Cohort of HIV Infected Patients Going in and out of the San Francisco County Jail

**DOI:** 10.1371/journal.pone.0007115

**Published:** 2009-09-22

**Authors:** Nitika Pant Pai, Milton Estes, Erica E. M. Moodie, Arthur L. Reingold, Jacqueline P. Tulsky

**Affiliations:** 1 Division of Clinical Epidemiology, Division of Infectious Diseases, McGill University Health Center, Montreal, Canada; 2 Forensic AIDS Project, Department of Public Health, San Francisco, California, United States of America; 3 Department of Epidemiology & Biostatistics, McGill University, Montreal, Canada; 4 Division of Epidemiology, University of California, Berkeley, California, United States of America; 5 University of California San Francisco, Positive Health Program, San Francisco General Hospital, San Francisco, California, United States of America; Lerner Research Institute, Cleveland Clinic, United States of America

## Abstract

**Background:**

Jails are an important venue of HIV care and a place for identification, treatment and referral for care. HIV infected inmates in the San Francisco County jail are offered antiretroviral treatment (ART), which many take only while in jail. We evaluated the effect of ART administration in a cohort of jail inmates going in and out of jail over a nine year period.

**Methodology/Principal Findings:**

In this retrospective study, we examined inmates with HIV going in and out of jail. Inmates were categorized by patterns of ART use: *continuous* ART - ART both in and out of jail, *intermittent* ART - ART only in jail; *never on* ART - eligible by national guidelines, but refused ART. CD4 and HIV viral load (VL) were compared over time in these groups. Over a 9 year period, 512 inmates were studied: 388 (76%) on intermittent ART, 79 (15%) on continuous ART and 45(9%) never-on ART. In a linear mixed model analysis, inmates on intermittent ART were 1.43; 95%CI (1.03, 1.99) times and those never on ART were 2.89; 95%CI (1.71, 4.87) times more likely to have higher VL than inmates on continuous ART. Furthermore, Inmates on intermittent ART and never-on ART lost 1.60; 95%CI (1.06, 2.13) and 1.97; 95%CI (0.96, 3.00) more CD4 cells per month, respectively, compared to continuously treated inmates. The continuous ART inmates gained 0.67CD4 cells/month.

**Conclusions/Significance:**

Continuous ART therapy in jail inmate's benefits CD4 cell counts and control of VL especially compared to those who never took ART. Although jail inmates on intermittent ART were more likely to lose CD4 cells and experience higher VL over time than those on continuous ART, CD4 cell loss was slower in these inmates as compared to inmates never on ART. Further studies are needed to evaluate whether or not intermittent ART provides some benefit in outcome if continuous ART is not possible or likely.

## Introduction

In the United States, HIV infection is an important health problem among jails and prisons [1]. Over 2 million individuals are incarcerated and a quarter of HIV-infected individuals are believed to pass through correctional facilities annually [2]. Due to the high proportion of HIV-infected individuals passing through correctional facilities, jails and prisons serve as entry points and are often the most consistent site of HIV care for marginalized populations [3]. However, HIV care in jails, which are usually local, county run facilities for persons charged, but not convicted or serving short sentences, is often minimal because stays are assumed to be short and a single person may be in and out of jail many times in a single year.

In the mid 1990's, HIV/AIDS accounted for the top three causes of death in the United States [1]. Since the introduction of potent combination anti-retroviral therapy (ART), a reduction in AIDS deaths nationally, including inmates in correctional settings, has been reported [4]. This reduction has been attributed to care and treatment of HIV-infected individuals in correctional facilities in accordance with the guidelines of the US Center for Diseases Control (CDC). According to the guidelines, care and treatment in correctional facilities includes prophylaxis for opportunistic infections and directly administered ART [5].

Although HIV care while incarcerated is legally protected, this guaranteed right to care has significant variations in implementation and is not ensured once an incarcerated person is released. Many persons taking ART in jail or prison are unable or unwilling to continue taking their ART medications outside the correctional institutional setting. Under these circumstances, whether or not to start or resume ART while incarcerated is a dilemma. Balancing concerns about developing resistance to ART and the safety of intermittent therapy and unplanned but predictable treatment interruptions for whatever reason with the high mortality of untreated HIV/AIDS has been a difficult task for a thoughtful jail clinician.

To date, few studies have evaluated the effects of antiretroviral treatment over time among jail inmates [6], [7], [8]. In these studies, the lack of continuity of care outside the jails and the high rates of re-incarceration have been associated with a lower likelihood of achieving the benefit of ART as measured by surrogate markers such as a gain in CD4 cell count and suppression of HIV viral load (VL) [7], [8]. These studies were limited by:

a) absence of comparison groups [7], [8] such as inmates continuing treatment outside of jails or inmates never on treatment; and b) shorter duration of observation (1–2 years). We conducted a retrospective cohort study of HIV positive inmates in the San Francisco county jail over a nine year period. Our study objective was to evaluate the benefit of ART administered in jails, as measured by its impact on CD4 and VL over time.

## Materials and Methods

### Study setting

The San Francisco City and County jail's Forensic AIDS Project (FAP) provides HIV counseling and testing services, HIV related primary care including examinations, diagnostic tests, prophylaxis for opportunistic infections, and ART [5]. All medications that are approved by the US FDA for HIV care are available through the jail pharmacy. HIV care, including prescription of ART, is guided by published national recommendations from the Department of Health and Human Services and the Centers for Diseases Control and Prevention. If inmates have a credible history of taking ART immediately prior to incarceration, the same drugs are continued on entry to jail until further evaluation takes place. The HIV Clinician is on call to consult with the admitting jail clinician regarding the prescription of ART. All medical care, including ART while in jail, is free and voluntary. The majority of new or modified ART are written by a single practitioner (ME), an experienced HIV specialist.

Since 1996, the San Francisco jail health has maintained an electronic database of the health care of all inmates that includes documented HIV status and records of all pharmacy, laboratory and clinician contacts with all inmates. When an inmate is booked into the jail, he/she receives a series of medical assessments. The initial intake history and physical findings are recorded by jail healthcare providers. If an inmate was previously in the jail, electronic records are retrieved and a history of medical problems outside the jail is recorded. All reports of medication intake outside the jail are cross-checked by contacting the dispensing pharmacy and community providers.

### Data collection

This retrospective cohort study was based on a review of the Forensic AIDS Project database over a nine year period. Individuals identified and documented as HIV positive during the period 1996–2005, enrolled in the Forensic Aids Project (FAP); and in jail more than once were reviewed for inclusion in the study. All data were collected by one person (NPP) and cross-checked by a second person (JPT). All data were de-identified and saved on a password protected file.

This study was conducted according to the principles expressed in the Declaration of Helsinki. The study was approved by the Institutional Review Board of UCSF. Since the study required chart review, informed consent was waived.

During the period, 1^st^ January 1996- 31^st^ July 2005, 1439 HIV-positive inmates were screened for eligibility. The following criteria were used to enroll the inmate: i) was jailed on at least two separate occasions during the nine year period; ii) was confirmed HIV-positive, and iii) was eligible to be offered ART based on FAP's criteria which reflected current national guidelines at the time; iv) had CD4 and HIV RNA levels measured at all follow up visits in jail; and v) had pharmacy notes, community providers notes, and triage notes available to corroborate claims of medication intake outside the jails.

Of 1439 inmates of FAP database screened for eligibility, 739 (51%) did not qualify for inclusion for the following reasons: i) in jail for very short durations (i.e. less than ten days), or ii) only one period of incarceration. Of the remaining 700 potentially eligible inmates, 69 were further excluded due to missing data (i.e., laboratory, clinical or pharmacy); and an additional 119 inmates were excluded who did not qualify for ART initiation either due to very high CD4 cell count or low VL and lack of symptoms. Thus, data were analyzed on a final sample of 512 inmates who were eligible for ART that qualified for inclusion in the study.

### Data Analysis

Data were abstracted in Microsoft Access and analyzed in R version 2.3.1 (open access software). Based on the patterns of medication intake, the 512 inmates were divided into three groups, i) *intermittent* ART group: inmates who took ART while in jail; ii) *continuous* ART group: inmates who took ART while in jail and after release from jail including the time between incarcerations; iii) *never on* ART group: inmates who at the time of triage qualified for ART but declined ART both inside and outside of jail.

Using linear mixed effects models in the three groups of inmates characterized by ART usage pattern, control of HIV infection was evaluated by examining the surrogate markers (i.e., VL and CD4 T cell counts). Linear mixed effects models allow the examination of variables that vary within and between individuals, and also take the correlation structure arising from information that is clustered within individuals into account [9], [10].

We first fitted a random intercepts model, which supposes a common association (slope) between CD4 cell counts and variables such as time or VL [10]. We then allowed the coefficients for the slopes to vary; this supposes that there is some between-person variability in the rate of change of CD4 over time, and some between person variability in the rate of change in VL over time [10]. The estimation procedure for both the random intercepts and the random intercepts and slopes models used Restricted Maximum Likelihood, as this is known to provide better estimates of standard errors than Maximum Likelihood [10]. Interaction and polynomial terms were also considered in the model but were not significant and were therefore not included in the final model.

The estimates of linear mixed effects models are interpreted like ordinary linear regression models. Referent group for comparison in models 2a, 2b is the continuous treatment group. Referent groups for variables Gender are Males and for Ethnicity are Caucasians.

### Changes in CD4 cell counts over time across treatment groups

The association measure is the mean difference in CD4 cell count between a treatment category and its reference. To assess the control of HIV infection in the ART group, we first considered the immunological outcome CD4 cell counts over time. The relationship between ART group and CD4 cell counts was investigated using linear mixed effects models. The possible confounders included in the model were age, ethnicity, gender, baseline CD4 cell count, and log transformed VL. VL was time–varying, while all other possible confounders were measured at baseline. Interactions of ART group and time were explored, as was a term for a polynomial of time (time^2^), as prior clinical knowledge suggested that interactions may be relevant.

Using 2807 measurements on the 512 inmates, the best-fitting model as determined by AIC included an interaction between ART group and time; the addition of squared-time was not deemed to be necessary. There was no evidence of between person variability in the changes in CD4 over time. We therefore report results from a random intercepts model. The rate of change in CD4 cell counts result from the inclusion of interactions between treatment group and time in months into the mean model. The estimates of the rate of change therefore are derived, for example, by combining the information from the rate of change amongst always-treated inmates (a gain of .67 cells) and the interaction of time with intermittent treatment (a loss of 1.60 cells) resulting in a net change of a loss of .93 cells.

### Changes in VL over time across treatment groups

The association measure is the median multiplicative VL between the treatment category and its reference. Using linear mixed models, we also examined log VL across the three ART groups. All models included age, race/ethnicity, gender, and CD4 cell count (which varied over time) As described above, we explored whether including an interaction term between ART group and time, or random slopes in time, improved the model fit. The interaction and the polynomial in time were not deemed necessary; however, allowing for between-person variability in the change in VL over time (i.e., a random slope) appeared to improve the model fit.

## Results

Over a nine year period of observation, the median follow-up duration for each inmate was 31 months (inter-quartile range: 6 months - 9.5 years). The 512 individuals had a median of 5 jail stays (range 2–20), and 36% had more than five visits in jail. The average jail stay length was 104 days (3.5 months) The median age of the inmates at study entry was 36 years (inter quartile range 19–66 years) (Table 1). A majority of the participants were African Americans (51%). Men accounted for 86% of the cohort. Inmates in the continuous ART group were the oldest. In all three groups of inmates, CD4 and VL were considered at baseline in jail. More than three-quarters (76%) of the inmates took intermittent ART; 9.0% refused ART and the remaining 15% took continuous ART throughout the study period. Using chi-square tests, we tested for differences amongst three ART groups (TABLE 1). At baseline, factors that were significant between the three groups are age, baseline VL (p<0.05). Some other significant factors from table 1 are follow up time in jail, exit CD4, exit VL (p<0.05) Over time, based on results of final models (Table 2, 3), the factors that were associated with category of ART included age, gender, ethnicity, time on treatment, baseline CD4 and baseline VL.

**Table 1:** Characteristics of study participants by treatment pattern: continuous, Intermittent and never on treatment.

	Continuous (*N = 79)	Intermittent (*N = 388)	Never on treatment (*N = 45)	P value
**Follow-up in jail, median (IQR), months**	38.2 (12.1–64.1)	39.5 (11.9–63.3)	26.3 (5.5–40.4)	0.018
**Age, median (IQR), Years**	37.6 (32.1–42.7)	35.3 (30.6–40.7)	34.9 (29.6–40.0)	0.045
**Ethnicity (%)**				
Caucasian	40.5	39.2	37.8	0.948
African American	49.4	51.3	48.9	
Other	10.1	9.5	13.3	
**Sex (%)**				
Male	91.1	86.6	73.3	0.086
Females	7.6	11.9	24.4	
Transgender	1.3	1.5	1.5	
**Baseline CD4, median(IQR)**	302 (185–448)	321.5(191–463)	309 (183–582)	0.678
**Exit CD4, median (IQR)**	361 (218–538)	290 (139–428)	261 (137–427)	0.017
**Baseline viral load, median (IQR)**	795(349–20,780)	7465 (500–40,200)	11260 (2,252–57,260)	<0.001
**Exit viral load, median (IQR)**	378 (75–4,584)	4,644 (105–32,260)	13,000 (3,247 –72,070)	<0.001

*N  =  sample size. Baseline CD4 and baseline viral load (VL) refer to the first available CD4 and VL in an inmate; Exit CD4 and Exit VL refer to the last available CD4 and VL on the inmate. P-values are derived from Kruskal-Wallis or chi-squared tests for continuous and categorical variables, respectively.

**Table 2:** Multivariate associations between demographic and clinical characteristics and CD4 cell count levels. Continuous treatments at study entry are the referent state. **Model 1: CD4.**

Characteristic	Estimate (95% CI)	p-value
**Treatment pattern (baseline mean)**		
Intermittent	10.18 (−20.20, 40.57)	0.512
Never on treatment	30.49 (−16.62, 77.59)	0.205
**Time (months)**	0.67 (0.18, 1.16)	0.007
**Treatment pattern×time**		
Intermittent×time	−1.60 (−2.13, −1.06)	<0.001
Never-on-treatment×time	−1.97 (−3.00, −0.94)	<0.001
* **Age**	0.05 (−1.29, 1.38)	0.944
**Gender**		
Female	2.73 (−27.77, 33.23)	0.861
Transgender	−2.03 (−86.31, 82.26)	0.962
**Ethnicity**		
African American	9.63 (−11.75, 31.02)	0.378
Other	8.85 (−28.07, 45.77)	0.639
**Baseline CD4**	0.79 (0.75, 0.83)	<0.001
**Viral load** †	−33.33 (−37.82, −28.83)	<0.001

Association measure is the mean difference in CD4 cell counts between a treatment category and its referent category.

†VL (log_10_ scale) is translated by log_10_ (5,000), and is time-varying.

*Age is translated (i.e., subtracted) by 30 years.

**Table 3:** Model 2: VL. Multivariate associations between demographic and clinical characteristics and viral load(VL). Continuous treatments at study entry are the referent state.

Characteristic	Estimate‡ (95% CI)	p-value
**Treatment pattern**		
Intermittent	1.34(0.92, 1.96)	0.133
Never on treatment	2.74 (1.50, 5.00)	0.001
**Time (months)**	0.99 (0.98, 1.00)	0.151
**Treatment pattern×time**		
Intermittent**×**time	1.00 (0.99, 1.02)	0.496
Never-on-treatment**×**time	1.00 (0.98, 1.03)	0.774
**Age**	0.98 (0.96, 0.99)	0.006
**Gender**		
Female	1.04 (0.72, 1.51)	0.826
Transgender	1.64 (0.57, 4.72)	0.357
**Ethnicity**		
African American	1.19 (0.93, 1.53)	0.172
Other	0.96 (0.62, 1.49)	0.857
**Baseline CD4**	1.00 (1.00, 1.00)	<0.001
**CD4**	1.00 (0.99, 1.00)	<0.001
**Baseline viral load** †	3.20 (2.86, 3.57)	<0.001

‡Estimates have been back-transformed to reflect a multiplicative change in VL (i.e., to reflect associated multiplicative changes on the natural scale rather than additive changes on the log scale) **Association measure is the median multiplicative VL between the treatment category and its referent state.**

†VL (log_10_ scale) is translated by log_10_ (5,000).

### Change in CD4 cell counts over time across treatment groups

There were strong associations between ART groups with CD4 cell counts and HIV VL over time consistent with the use of guidelines to offer ART for lower CD4 cell counts and higher VL. The interaction terms for ART groups and time were significant for intermittent ART and never on ART groups. (Table 2)

On an average, continuously treated inmates gained an average adjusted of 0.67 CD4 cells per month. The difference (95% CI) in the adjusted rate of change in the intermittently treated inmates as compared to continuously treated inmates was −1.60 (−2.13, −1.06), resulting in the intermittent ART group inmates lost CD4 cells at an average adjusted rate of 0.93 cells per month. The difference (95% CI) in the adjusted rate of change in the never on ART inmates as compared to continuously treated inmates was −1.97 (−3.00, −0.96), so that the never-treated inmates lost CD4 cells at an average adjusted rate of 1.29 cells per month. However, this difference was not statistically significant. (p = 0.33). At baseline, the differences in CD4 counts and VL among the three ART groups were statistically significant (p<0.001; Table 2)

As observed in table 2, significant differences in the rate of change of treatment groups were observed over time (variables intermittent* time, never on treatment*time; as indicated by p values<0.001). Variable time (months) was significant (p value <0.007). Further interpreting other significant variables like age in the models, if we were to compare two inmates who were the same with respect to treatment group, time since baseline, ethnicity, and gender but differed in age by 1 year, we would expect the CD4 cell counts to differ by 0.05 (−1.29, 1.38) cells. Furthermore, if we were to compare two inmates who were the same with respect to treatment group, time since baseline, ethnicity, and age but of different gender ( female vs. male), then, we would expect CD4 cell count to differ by 2.73 (−27.77, 33,23) cells.

**Figure 1:**
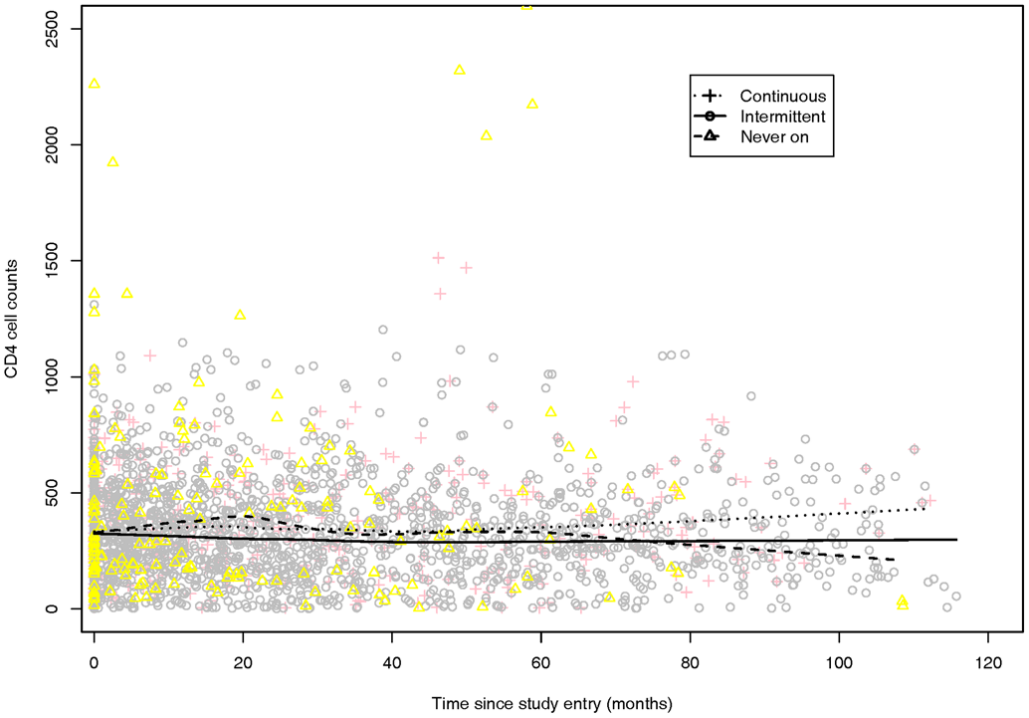
Local average of CD4 counts over time by treatment pattern: intermittent, continuous, and never-on treatment. In the figure, a Continuous ART inmate is noted with pink plus (dotted line), and Intermittent ART inmate is noted with a gray circle (solid line), and the yellow triangles (dashed line) mark the Eligible but Never on treatment inmates.

**Figure 2:**
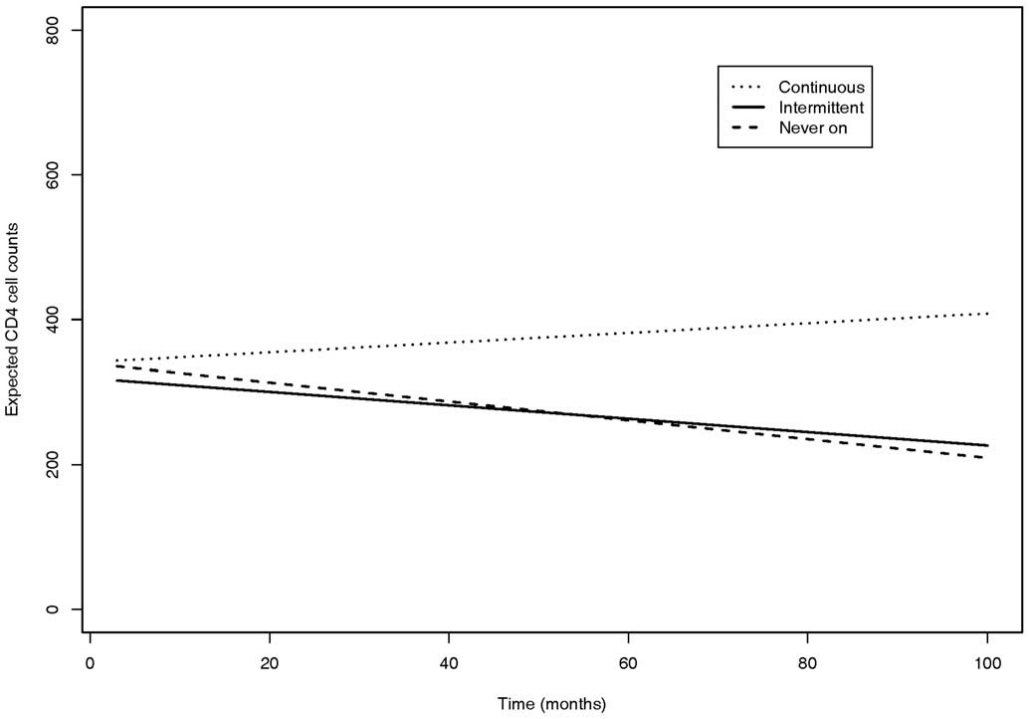
Expected CD4 over time by for a 30 year-old Caucasian male, as predicted from a linear mixed effects model. Baseline CD4 counts and viral load (VL) over time are taken to reflect the baseline status of the inmates by the pattern of medication intake: CD4 cells at 330 cells/ml at baseline and a VL of 800 copies/ml for an inmate who was continuously treated; CD4 cells at 330 cells/ml at baseline and a VL of 7,500 copies/ml for an inmate who was intermittently treated; CD4 cells at 430 cells/ml at baseline and a VL of 8,000 copies/ml for an inmate who was never treated.

Figure 1 and Figure 2 illustrates the un-adjusted and adjusted changes in CD4 cell counts for the three ART groups. In Figure 1, the CD4 cell counts increase in the continuous ART group and fall most rapidly in the never on ART inmates. In Figure 1, a descriptive summary of the data is presented and the VL is not held constant (unadjusted).

In Figure 2, we have attempted to more clearly illustrate the effect of each ART group. VL was held constant in deriving these predicted trajectories (adjusted). The baseline CD4 and the VL were selected to reflect typical (baseline) values for each of the ART groups. The estimated course of CD4 cell count over time predicted by the model for a 30 year old male with a constant VL is shown. Note the trajectories in each of the ART groups in Figure 2 are broadly similar to the observed trajectories in Figure 1. We would not expect these to coincide exactly since VL was held constant over time in Figure 2, which is not the case in the unadjusted, descriptive summary of the data shown in Figure 1. These results suggest that compared to inmates that were on never on ART, the inmates in the continuous ART group documented the best response overall in terms of gain in CD4 cells over time, followed by the inmates in the Intermittent ART group with a slower rate of loss of CD4 cells over time.

### Changes in HIV VL over time across treatment groups

Overall the HIV VL decreased by 6% per year in each of the three treatment groups. From the model, (Table 3) we can infer that the VL of intermittent ART inmates was approximately one and a half times and of never-on ART inmates three times greater than those on continuous ART. After adjusting for the different covariate distributions, although there was significant differences in the change in HIV VL over time between Never on treatment group and referent continuous treatment group, (2.74 (1.50, 5.00); p value <0.001), these differences between intermittent treatment and continuous treatment group were non significant (1.34 (0.92–1.96); p value <0.133).Furthermore, Baseline CD4, Baseline VL, and CD4 over time, were found to significantly differ between treatment groups (p<0.05) and a non- significant effect of time was also observed (0.99(0.98,1.00) p value<0.151).

**Figure 3:**
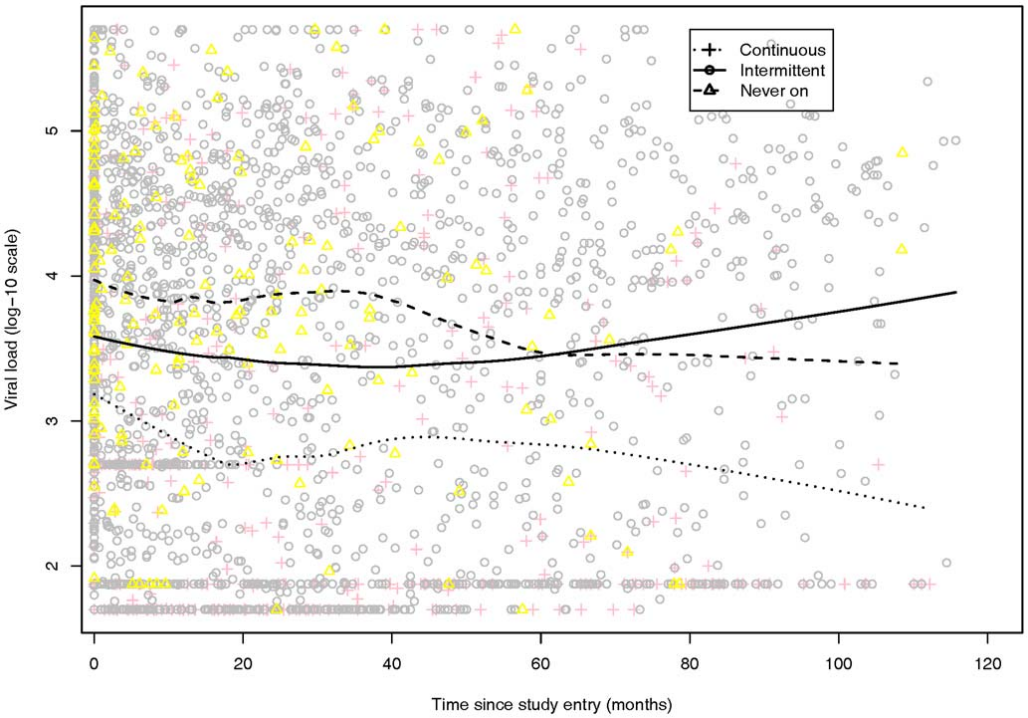
Local average of viral load (VL) over time by treatment pattern: intermittent, continuous, and never-on treatment. In the figure, a Continuous ART inmate is noted with pink plus (dotted line), and Intermittent ART inmate is noted with a gray circle (solid line), and the yellow triangles (dashed line) mark the Eligible but Never on treatment inmates.

In figure 3, as in figure 1, the un-adjusted local average observed trajectories of VL in three treatment groups have been illustrated.

## Discussion

This is the first study to examine the effect of ART on a cohort of HIV infected persons going in and out of a county jail over a relatively long period of time. Our results indicate that a majority of inmates (76%) interrupted ART after being released from jail. A few inmates managed to stay on medications (15%), and a minority (9%) although eligible for ART never agree to take ART, whether in or out of jail. Results from linear mixed models suggest that inmates on intermittent and never on ART reported a loss of CD4 cells at an average adjusted rate of 0.93 cells and 1.29 cells/month respectively. In comparison, and as expected, the continuous ART group gained CD4 cells at an average adjusted rate of 0.67 cells per month. Similarly, those on intermittent ART and never on ART reported higher VL (i.e., 1.5 times and 3 times) in comparison with inmates on continuous ART.

### How do our study findings compare with those of previous studies?

An examination of Table 1 suggests that there were no racial disparities across ART groups. This is in contrast with data from previous studies that suggest that African Americans disproportionately lack adequate care [11], [12]. In our study, inmates on continuous ART were demographically similar to inmates on intermittent ART. All inmates taking ART were offered the same standard of care in jails.

In another study by Springer et al, 1866 prison inmates were followed for mean 478 day period. Twenty-seven percent of re-incarcerated inmates lost the beneficial effects of ART. However 59% achieved an undetectable HIV VL (VL<400 copies/ml) by their last visit in prison [7]. In another study on 15 prison inmates by Stephenson et al., over 38% of re-incarcerated inmates lost the benefit of ART, with deleterious effects on VL and CD4 after release over a two year period [8]. In comparison, in our study encompassed information from a nine year period, with a median follow up of 31 months (2.6 years), 169 (32.4%) inmates achieved viral suppression at their last visit in jail. This benefit was present across both the continuous and intermittent treatment groups.

Some aspects of our study results are pertinent in evaluating the approach to care and treatment in jailed HIV infected inmates. Although a majority (76%; 388/512) of inmates were on intermittent ART demonstrating the difficulty of maintaining adherence to their HIV medications outside of jail, many inmates managed to continue their ART over time (15%; 79/512). Intermittent ART lost CD4 over time, and had higher VL suggesting loss of beneficial effects of ART compared to continuous ART. On the other hand, there is a suggestion that intermittent ART provides some benefit in comparison to never on ART.

We are concerned about deleterious effects of intermittent therapy in light of SMART (Strategies for Management of ART) data and the possibility of the development of resistance [13]. SMART, however, compared only intermittent CD4 cell-guided ART interruptions with continuous ART [13], [14]. The reality of HIV care for most incarcerated patients at present is intermittent treatment or no treatment at all. In any jail study, the comparison of individuals on intermittent ART is further complicated due to lifestyle factors affecting adherence and co-morbidities. In our cohort analysis, it is clear that continuous ART is best; however those who are on intermittent therapy are better off than the never on ART. Medications must and should be provided, and the platforms for provision of continuous care outside jails should be focused on maintaining the benefits of ART after release.

Our study reflects a true natural history study of a large cohort of incarcerated HIV-infected persons. It highlights the need to support continuous ART therapy and the importance of continuity of care services for HIV infected persons who enter the cycle of incarceration. Given the recidivism rates of 60%–70% jail settings must be optimized as intermittent care sites with seamless connections to community care.

This is the first study in a jail cohort to apply multi-level analyses. It will be inappropriate to compare this data with a standard cohort study or a compliant trial population, but it does raise important questions that should be addressed by prospective evaluations of similar incarcerated populations. Although questions on adherence and resistance are compelling, they cannot be answered in this context, since this study was aimed to be a preliminary exploration of the natural history of a jail cohort. Funding future studies on factors associated with staying on medications outside jail will aid in understanding individual patient and system issues that impact continuity with ART outside jails.

### Strengths

This large retrospective cohort study spans an observation period of 9 years, and attempts to evaluate the impact of antiretroviral treatment in the management of HIV infection over this long period of time. The effects of ART have been compared across three groups of inmates characterized by patterns of ART usage (i.e., intermittent ART, continuous ART, and never on ART). The size of the inmate cohort is large, and data were verified from multiple sources (i.e., pharmacy, laboratory, community providers), increasing confidence in their reliability. Furthermore, claims of medication intake in the community were ascertained by linked pharmacy and laboratory records and from the network of community providers.

### Limitations

Our study was limited by the nature of retrospective cohort jail data available. The study did not address factors that might predispose to non-adherence or adherence of ART such as repeated incarceration itself, unstable housing, mental illness, and drug and alcohol dependency. Categorization into the 3 groups was based on chart review data that was drawn from multiple sources. We were unable to examine the development of viral resistance over time; although it is possible that viral resistance in the intermittent group was unusual, reflecting the abrupt discontinuation of ART. It is possible that the never on ART Group included individuals with low T-cells who did not progress, thus making them seem healthier. Hospitalization rates were not measured, nor were concomitant illnesses or mortality rates. We believe that future studies of this important population would benefit from analyzing these outcomes.

### Conclusion

The dual epidemics of incarceration and HIV in the US have led to a high concentration of HIV -infected individuals in incarcerated settings [15]. With HIV testing guidelines being expanded to all populations, it is likely that more HIV infection will be detected in incarcerated individuals in the future. This study demonstrates that there is a clear benefit from continuous ART therapy in a group of persons going in and out of jail on both CD4 cell counts and VL especially compared to those who refuse ART despite eligibility by national treatment guidelines. There was also no clear evidence in this cohort of harmful effects on CD4 and VL for ART therapy taken only in jail. There is a need to examine ART policies both inside and outside correctional settings and aim towards the establishment of effective life long management of HIV infection for persons affected by incarceration. To maintain the benefit of ART outside jails, effective community transition and prison release programs that focus on ART management along with linkages to community providers, stabilization of housing, and community based support services are needed [2], [16].
